# Maternal alexithymia and caregiving behavior: the role of executive functioning - A FinnBrain Birth Cohort study

**DOI:** 10.1007/s00737-024-01523-4

**Published:** 2024-11-05

**Authors:** Elisabeth Nordenswan, Kirby Deater-Deckard, Eeva-Leena Kataja, Mira Karrasch, Matti Laine, Juho Pelto, Eeva Holmberg, Hetti Lahtela, Hanna Ahrnberg, Jani Kajanoja, Max Karukivi, Hasse Karlsson, Linnea Karlsson, Riikka Korja

**Affiliations:** 1 Department of Clinical Medicine, The FinnBrain Birth Cohort Study, Turku Brain and Mind Center, University of Turku, Turku, Finland; 2 Department of Psychology and Speech-Language Pathology, University of Turku, Turku, Finland; 3 The Centre of Excellence for Learning Dynamics and Intervention Research (InterLearn), University of Turku, Turku, Finland; 4 Helsinki Collegium for Advanced Studies, Helsinki, Finland; 5 Department of Psychological and Brain Sciences, University of Massachusetts Amherst, Amherst, MA USA; 6 Centre for Population Health Research, Turku University Hospital and University of Turku, Turku, Finland; 7 Department of Psychology, Åbo Akademi University, Turku, Finland; 8 Department of Mathematics and Statistics, University of Turku, Turku, Finland; 9 Department of Psychiatry, Turku University Hospital and University of Turku, Turku, Finland; 10 Department of Adolescent Psychiatry, Turku University Hospital and University of Turku, Turku, Finland; 11 Department of Clinical Medicine, Paediatrics and Adolescent Medicine, Turku University Hospital and University of Turku, Turku, Finland; 12 Department of Clinical Medicine, Unit of Public Health, University of Turku, Turku, Finland

**Keywords:** Alexithymia, Caregiving behavior, Early parenthood, Emotional availability, Executive functioning

## Abstract

**Purpose:**

The growing interest in parental cognition calls for research clarifying how cognition interacts with other parenting determinants to shape caregiving behavior. We studied the interplay between executive functioning (EF; cognitive processes that enable goal-directed thinking and behavior) and alexithymic traits (characterized by emotion processing/regulation difficulties) in relation to emotional availability (EA; the dyad’s ability to share an emotionally healthy relationship). As EF has been reported to shape parents’ ability to regulate thoughts and emotions during caregiving, we examined whether EF moderated the association between maternal alexithymic traits, and EA.

**Methods:**

Among 119 mothers with 2.5-year-olds drawn from the FinnBrain Birth Cohort, EF was measured with Cogstate tasks, alexithymic traits with the Toronto Alexithymia Scale (TAS-20), and caregiving with the Emotional Availability Scales (EAS).

**Results:**

More alexithymic traits on the TAS-20 subscale Externally Oriented Thinking (EOT) were associated with poorer caregiving in a hierarchical regression analysis (Δ*R*^2^ = 0.05, *p* = .01). A marginally significant moderation effect was found when adding the EOTxEF interaction term to the model (Δ*R*^2^ = 0.03, *p* = .06). These associations weakened slightly when controlling for education level. Estimation of simple slopes and a Johnson-Neyman figure indicated a significant association between higher EOT and lower EAS, that increased in strength as EF decreased from the group mean level.

**Conclusions:**

The influence of cognitive alexithymic traits on EA could be especially pronounced among low EF parents, but further studies are needed to support and extend the findings. The potential role of parental reflective functioning in this context is discussed.

**Supplementary Information:**

The online version contains supplementary material available at 10.1007/s00737-024-01523-4.

Cognitive changes have been reported in relation to pregnancy and early parenthood (McCormack et al. 2023), but there is limited understanding for how these changes influence parental caregiving behavior. As caregiving is shaped by a multitude of parenting determinants (Bornstein 2016), knowledge about how these determinants interact is central. Specifically, it is unknown how parental cognition and personality, a well-known determinant of caregiving behavior (Prinzie et al. 2009), interact in relation to parenting. Considering the central role of parenting in early child development (Nelson et al. 2019), uncovering the dynamics between parental cognitive functioning, personality, and caregiving behavior is especially important in early parenthood.

Knowledge about the role of parental cognition in caregiving behavior is limited. Accumulating evidence has linked parental executive functioning (EF) with caregiving behavior (Crandall et al. 2015). EF includes cognitive processes that enable goal-directed thinking and behavior, consisting of working memory, set-shifting, and inhibitory control that together support higher-order problem-solving and planning (Friedman and Miyake 2017). EF enables taking time to think before acting, resisting impulses, staying focused, thinking through ideas, and meeting novel challenges (Diamond 2013). As caregiving requires parents to constantly learn and adapt, it is plausible that parental EF would influence caregiving. Evidence suggests better EF predicts more sensitive, responsive caregiving, while poorer EF is linked to harsher caregiving and risk for child maltreatment (Crandall et al. 2015).

Emotional availability (EA; Biringen et al. 2014) is an important aspect of caregiving that may be related to EF. EA describes the parent-child dyad’s ability to share an emotionally healthy relationship. EA is rooted in attachment theory, and includes parental sensitivity, structuring, non-intrusiveness and non-hostility. EA has been linked to child emotion regulation, social competence, language skills, and internalizing/externalizing problems (Saunders et al. 2015). Knowledge about determinants of EA is needed to effectively support families who struggle with their parent-child relationships. A few studies have examined parental EF and EA. Harris and colleagues (2021), and Nordenswan and colleagues (2021), found that higher EF was modestly associated with more EA in the general population, while Porreca and colleagues (2018) reported moderate EF-EA associations among mothers with substance use disorder. Thus, EF may be a key factor that shapes EA, and this process may vary in different populations.

Parental EF may also moderate how other determinants shape caregiving behavior. For example, Deater-Deckard and colleagues (2012) found child conduct problems to be associated with harsh parenting only among mothers with lower EF, suggesting that parental EF supports regulating negative emotions arising from challenging child behavior. Similarly, Sturge-Apple and colleagues (2014) found maternal working memory to moderate the association between child-oriented attributions and harsh discipline, perhaps because working memory helps mothers disaggregate parenting behavior from negative attributions. Thus, EF plays a role in regulation of caregiver thoughts and emotions. As emotion regulation strategies are linked with personality traits (Baranczuk 2019), and personality is a central determinant of caregiving (Prinzie et al. 2009), research on the interaction between parental cognition and personality in relation to caregiving is needed.

When examining the interplay between parental cognition and personality in relation to caregiving, there are many personality traits to consider. The personality construct alexithymia includes a decreased ability to identify and verbalize emotions, a limited imaginative capacity, and an externally oriented thinking style, i.e., thinking pragmatically and lacking interest in emotional experiences and introspection (Sifneos 1973). By hindering emotion regulation, alexithymia predisposes individuals to somatic and psychological symptoms (Kajanoja 2019). Research on parental alexithymia has uncovered links with parenting behavior. More specifically, higher levels of alexithymic traits are associated with less parental mentalizing and reflective functioning, i.e., understanding the child’s mind and showing interest in the child’s internal states (Ahrnberg et al. 2020). Alexithymic traits have furthermore been linked with authoritarian and permissive parenting styles (Cuzzocrea et al. 2015), and with poorer mother-child interaction quality (Ahrnberg et al. 2021; Porreca et al. 2020; Yürümez et al. 2014). EF plays a central role in emotion regulation (Zelazo and Cunningham 2007), so it seems likely that parental EF could influence how alexithymic traits shape caregiving. To our knowledge, the joint effects of parental alexithymic traits and EF on caregiving have not been estimated.

The current study examined associations between maternal alexithymic traits and EA, and whether these associations were moderated by maternal EF. Based on the literature, *we expected higher levels of alexithymic traits to be associated with lower EA.* Also, *we expected the alexithymic traits/EA associations to be strongest among mothers with lower EF*,* and weakest among those with higher EF*.

## Materials and methods

### Participants

Participants (*N* = 119 general population mothers) were from the FinnBrain Birth Cohort Study (Karlsson et al. 2018). Pregnant women in South-West Finland were recruited to the cohort 2011–2015. Inclusion criteria were sufficient knowledge of Finnish or Swedish and a normal screening result. The main data for this study was collected within FinnBrain’s Child Development and Parental Functioning Lab. Recruitment to the lab’s study visits was primarily based on participation in the lab’s prior visits. See Online Resource 1 for details on participants.

### Procedures

The Ethics Committee of the Hospital District of Southwest Finland approved the protocol. Written informed consent was obtained from the mothers. Alexithymic traits were assessed with a questionnaire sent home to the participants at six months postpartum. Maternal EF and EA were assessed during two separate study visits, conducted in quiet examination rooms at FinnBrain’s research facilities when the participants’ children were 2.5 years old. Maternal EF was measured with computerized neuropsychological tasks, presented under supervision on a laptop. Maternal EA was assessed with a 15–20 min. long video-recorded free-play situation. The mothers and their toddlers sat on a comfortable floor-carpet, and age-appropriate toys were provided.

### Measures

*Executive functioning* was measured with five Cogstate (www.cogstate.com) EF tasks: Two-Back (TWOB), Set-Shifting (SETS), Groton Maze Learning (GML), Continuous Paired Associate Learning (CPAL), and International Shopping List (ISL). The task scores were standardized and combined into an EF mean score, which was re-standardized (see the Online Resource 1 for more details on all measures).

*Caregiving behavior* was assessed with the Emotional Availability Scales (EAS; Biringen 2008), which operationalize a parent-child dyad’s capacity to share an emotionally healthy relationship (Biringen et al. 2014). The four parental EAS dimensions were combined into an EAS mean score where higher values indicate better EA, i.e., more sensitivity, more structuring, less intrusiveness, and less hostility.

*Alexithymic traits* were self-reported with the Toronto Alexithymia Scale (TAS-20; Bagby et al. 1994a; Bagby et al. 1994a). The TAS-20 sum score and subscales difficulty identifying feelings (DIF), difficulty describing feelings (DDF), and externally oriented thinking (EOT) were included in the analyses. The EOT subscale was standardized prior to its inclusion in an EOTxEF interaction term.

### Analytic approach

All analyses were performed with SPSS (version 26). Variables were evaluated for normality and descriptive statistics were calculated. Some mothers had previously completed Cogstate testing, so practice effects were controlled for by comparing the first-time participants’ results (*n* = 93) with the re-tested participants’ results (*n* = 26) using Mann–Whitney *U* tests. Scatter plots were examined to explore the linearity of the associations between variables and to identify potential outliers. Bivariate correlations were calculated. Because the TAS-20 subscales previously have shown different associations with the EAS (Ahrnberg et al. 2021), the TAS-20 total score and the three subscales were examined separately in the bivariate correlations. Of the TAS-20 variables, only EOT was significantly associated with the EAS. Consequently, only EOT was included in the multiple regression analyses that tested the study hypotheses. Similarly, education level was the only covariate significantly associated with the EAS, and was therefore included in the regression analyses (results are reported with and without it).

The interaction effects of the regression models were further explored by estimation of simple slopes. TAS-20 EOT was utilized as the independent variable, the EAS composite as the dependent variable, and the EF composite as the moderator. The simple slopes were estimated at the mean, and at 1 *SD*, 1.5 *SD* and 2 *SD* above and below the mean of the moderator. The pattern of the moderation effect across the full range of the moderator was illustrated in a Johnson-Neyman plot (Bauer et al. 2005).

## Results

### Descriptive statistics

See Table 1 for the descriptive statistics. Participants’ EF levels were typical of community sample participants. The TWOB and GML group mean scores were within the normal range (± 1 *SD*) of normative data for the healthy adult age groups 18-34yrs and 35-49yrs, while the ISL results were slightly better than for both normative age groups. More errors were made on the CPAL than expected based on the norms. However, the CPAL normative sample is very small (18-34yrs *N* = 62, 35-49yrs *N* = 9) and should thus be viewed with caution. The EF scores of mothers who completed Cogstate once versus twice did not differ (*U* tests, *p* = .076-0.801). The participants reported low levels of alexithymia: 8.4% of the mothers reported TAS-20 total scores indicating moderate alexithymia, while only 1.7% reported scores signifying high alexithymia. The EAS scores reflected mostly positive, emotionally available caregiving behavior. The majority of the free-play situations were coded as good enough EA, as seen in Table 2.

**Table 1:** Descriptives of the study variables

Variable	Mean	Standard deviation	Range	
*Cogstate Tasks*				
GML	8.28	3.36	1–19	
CPAL	12.70	8.38	0–40	
ISL	8.08	1.59	5–11	
TWOB	1.31	0.13	1.03–1.57	
SETS	1.18	0.11	0.92–1.33	
EF composite	0	1	-2.16-2.32	
*Emotional Availability Scales*				
EAS composite	5.70	0.91	3.25-7.00	
Sensitivity	5.13	1.12	2–7	
Structuring	5.24	1.22	2–7	
Non-Intrusiveness	5.92	1.22	3–7	
Non-Hostility	6.53	0.70	4–7	
*Alexithymia Questionnaire*				
TAS-20 sum score	39.89	9.10	23–64	
DIF	11.97	4.73	7–26	
DDF	9.87	3.51	5–20	
EOT	18.06	4.13	9–29	

*Note* GML = Groton Maze Learning, CPAL = Continuous Paired Associate Learning, ISL = International Shopping List, TWOB = Two-Back, SETS = Set-Shifting, TAS-20 = The 20-item Toronto Alexithymia Scale, DIF = Difficulty Identifying Feelings, DDF = Difficulty Describing Feelings, EOT = Externally Oriented Thinking. The variables are described in more detail in the *Measures* section as well as in Online Resource 1

**Table 2:** Emotional availability scales results grouped according to EA Level

EAS dimension	Good enough	Somewhat problematic	Detached	Highly problematic
Sensitivity	47.9%	39.5%	11.8%	0.8%
Structuring	48.7%	41.2%	9.2%	0.8%
Non-Intrusiveness	68.1%	26.1%	5.9%	0.0%
Non-Hostility	92.4%	7.6%	0.0%	0.0%

*Note* The EAS dimensions are described in Online Resource 1

### Correlation results

Bivariate correlations are presented in Table 3. As expected, higher levels of alexithymic traits were significantly associated with lower EAS. However, this was only found for the TAS-20 subscale EOT. Three of the four EA scales, i.e., Sensitivity, Structuring, and Non-Hostility (but not Non-Intrusiveness) had very similar associations with TAS-20 EOT as the EAS composite, so we proceeded focusing on the EAS composite as the dependent variable in subsequent regression analyses. EF did not correlate significantly with the TAS-20 variables or the EAS. Of the potential covariates (i.e., participant age, number of children, education level, and WAIS-IV VCI), only education level was significant with the EAS composite. Thus, it was the only covariate included in the subsequent regression analyses.

**Table 3:** Bivariate correlations between the study variables

Variable	1.	2.	3.	4.	5.	6.	7.	8.	9.	10.	11.	12.	13.	14.
**1. EA Composite**	1													
**2. Sensitivity**	0.93**	1												
**3. Structuring**	0.89**	0.89**	1											
**4. Non-Intrusiveness**	0.80**	0.60**	0.50**	1										
**5. Non-Hostility**	0.78**	0.62**	0.58**	0.59**	1									
**6. TAS-20 Total Score**	− 0.14	− 0.17†	− 0.22*	− 0.01	− 0.06	1								
**7. DIF**	0.00	− 0.05	− 0.10	0.10	0.09	0.79**	1							
**8. DDF**	− 0.08	− 0.10	− 0.16	− 0.03	0.04	0.81**	0.59**	1						
**9. EOT**	− 0.23*	− 0.23*	− 0.24*	− 0.08	− 0.26*	0.61**	0.10	0.27**	1					
**10. EF Composite**	0.15	0.11	0.16†	0.09	0.16†	0.00	0.11	− 0.10	− 0.04	1				
**11. Age**	0.02	− 0.01	0.01	0.00	0.08	− 0.10	− 0.10	− 0.05	− 0.07	− 0.09	1			
**12. Parity**	0.12	0.08	0.12	0.06	0.10	− 0.05	0.03	− 0.05	− 0.06	0.21*	0.42**	1		
**13. Education level**	0.19*	0.18†	0.18†	0.12	0.22*	− 0.21*	− 0.00	− 0.15	− 0.30**	0.01	0.32**	0.17†	1	
**14. WAIS-IV VCI**	0.13	0.15	0.17	0.05	0.03	− 0.09	0.05	− 0.07	− 0.19†	0.23*	0.29**	0.10	0.42**	1

*Note* For correlations involving the variables Parity or Education Level, Spearman’s rho was used, for other correlations Pearson’s *r* was used. EA = emotional availability, TAS-20 = The 20-item Toronto Alexithymia Scale, DIF = difficulty identifying feelings, DDF = difficulty describing feelings, EOT = externally oriented thinking, EF = executive functioning, WAIS-IV VCI = The Wechsler Adults Intelligence Scale – Fourth Edition, verbal comprehension index

### Regression results

As only the link between the TAS-20 subscale EOT and the EAS composite was significant in the bivariate correlations, we focused on this link to further explore the association and test for a moderating effect of EF using hierarchical regression (once with education level covariate either excluded or included), with the following order of entry: TAS-20 EOT; EF; then EF x TAS-20 EOT. Results are presented in Table 4 (without education covariate) and Table 5 (with education covariate).

When education level was excluded, higher levels of EOT were significantly associated with lower EAS (Δ*R*^2^ = 0.05, *p* = .013). With education included, the effect of EOT weakened slightly (Δ*R*^2^ = 0.03, *p* = .046).

EF was not a significant predictor either with or without education included as a covariate. The EF x TAS-20 EOT interaction term was marginally significant regardless of whether education level was excluded (Table 4, Step 3: Δ*R*^2^ = 0.03, *p* = .063) or included (Table 5, Step 4: Δ*R*^2^ = 0.03, *p* = .065).

**Table 4:** First hierarchical multiple regression analysis excluding the covariate education: Effect of externally oriented thinking and executive functioning on the emotional availability scales composite

	*R* ^2^	*R*^2^∆	F∆	F∆ *p*-value	B	β	t	B *p*-value	B, 95.0% Confidence Interval	sr^2^
**Step 1**: EOT	0.05	0.05	6.36	0.013	− 0.06	− 0.23	-2.52	0.013	− 0.10/-0.01	0.05
**Step 2**: EF	0.07	0.02	2.39	0.125	0.14	0.14	1.55	0.125	− 0.04/0.32	0.02
**Step 3**: EF x EOT	0.10	0.03	3.52	0.063	0.04	0.80	1.88	0.063	− 0.00/0.09	0.03

*Note* EOT = externally oriented thinking, EF = five-task-executive functioning composite, EF x EOT = interaction term between EOT and EF. The variables are described in the *Measures* section, as well as in Online Resource 1

**Table 5:** Second hierarchical multiple regression analysis including the covariate education: Effect of externally oriented thinking and executive functioning on the emotional availability scales composite

	*R* ^2^	*R*^2^∆	F∆	F∆ *p*-value	B	β	t	B *p*-value	B, 95.0% confidence interval	sr^2^
**Step 1**: Education	0.03	0.03	3.87	0.052	0.22	0.18	1.97	0.052	-1.00/0.04	0.03
**Step 2**: EOT	0.07	0.03	4.08	0.046	− 0.05	− 0.19	-2.03	0.046	− 0.09/-0.00	0.03
**Step 3**: EF	0.08	0.01	1.79	0.184	0.12	0.12	1.34	0.184	− 0.06/0.30	0.01
**Step 4**: EF x EOT	0.11	0.03	3.47	0.065	0.04	− 0.17	1.86	0.065	− 0.00/0.09	0.03

*Note* EOT = externally oriented thinking, EF = five-task-executive functioning composite, EF x EOT = interaction term between EOT and EF. The variables are described in the *Measures* section, as well as in Online Resource 1

### Estimation of simple slopes and Johnson-Neyman figure

**Fig. 1:**
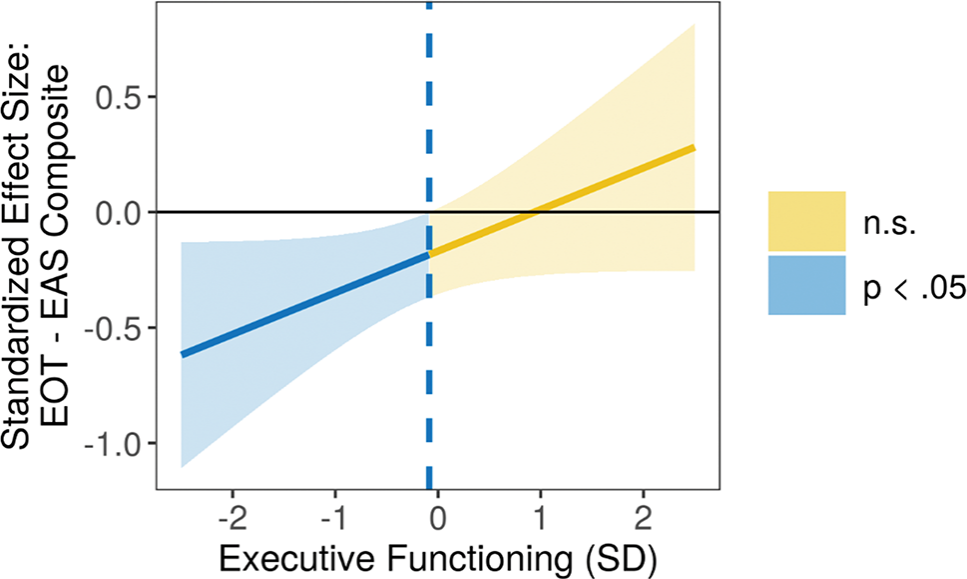
Effect size of externally oriented thinking on emotional availability composite, as moderated by executive functioning. *Note* The blue-and-yellow (dark-and-light) line depicts the regression coefficient of the TAS-20 subscale EOT on the EAS composite, as moderated by the EF composite (and including the covariate education level). The regression coefficient is statistically significant when the colored area is below zero on the X-axis. The association between EOT and the EAS composite is significant for mothers with EF values below the group mean, but non-significant for mothers with EF levels above the group mean

To probe the two-way interaction term, post-hoc simple slopes analyses revealed that the TAS-20 EOT/EAS association was different for mothers whose EF levels were below the group mean level vs. mothers with EF levels above the group mean level. In mothers with EF levels below the group mean, a significant association between higher TAS-20 EOT scores and lower EAS emerged, increasing in strength as EF levels decreased (1 *SD* below the mean: β = − 0.38, *p* = .002; 1.5 *SD* below the mean: β = − 0.47, *p* = .004; 2 *SD* below the mean: β = − 0.56, *p* = .006). In contrast, for mothers with EF levels above the group mean, TAS-20 EOT and EAS were not significantly associated (1 *SD* above the mean: β = − 0.02, *p* = .910; 1.5 *SD* above the mean: β = 0.08, *p* = .680; 2 *SD* above the mean: β = 0.17, *p* = .462). These results are in line with our hypothesis: alexithymic traits would be most strongly associated with caregiving behavior at lower levels of EF, and higher levels of EF would buffer this association. The interaction effect also is displayed in a Johnson-Neyman plot (Fig. 1).

## Discussion

We examined the association between alexithymic traits and emotional availability (EA) in caregiving behavior among general population mothers with 2.5-year-old children, and whether this association was moderated by maternal executive functioning (EF). In line with our hypotheses, we found an association between more alexithymic traits (measured with the 20-item Toronto Alexithymia Scale, TAS-20) and lower emotional availability (measured with the Emotional Availability Scales, EAS). Only the TAS-20 subscale externally oriented thinking (EOT) was associated with emotional availability. We also found a marginally significant moderating effect of maternal EF on the link between externally oriented thinking and emotional availability, showing that this link was significant only for mothers with lower EF.

The finding that higher levels of cognitive aspects of alexithymic traits were associated with lower emotional availability is consistent with prior research (Ahrnberg et al. 2021; Porreca et al. 2020). However, only the cognitive dimension EOT was linked with emotional availability. The other more affective dimensions of alexithymia (DIF; DDF) have consistently been associated with psychiatric symptomatology, while EOT has been associated with detached social behavior and low empathy (Grabe et al. 2004; Grynberg et al. 2010; Kajanoja et al. 2017; Kajanoja 2019; Vanheule et al. 2011). It seems plausible that the affective and cognitive dimensions of alexithymia could relate differently to caregiving. EOT is characterized by unemotional non-introspective cognition, and could impair awareness of the child’s emotions and mental states and parental emotional availability.

The link between the cognitive aspects of alexithymia and emotional availability was significant only among mothers with lower EF. Lower EF may make parents more vulnerable to influences of EOT on emotional availability. This aligns with evidence that burdensome caregiving determinants are more strongly associated with negative parenting for mothers with lower EF (Deater-Deckard et al. 2012; Sturge-Apple et al. 2014). Better EF likely enables parents to pause and think before reacting, making it easier to suppress reactions linked to an externally oriented thinking style and instead adapting to the child’s emotional needs.

Our findings also connect to research on parental mentalizing and reflective functioning. Higher levels of alexithymic traits have been associated with lower parental reflective functioning, and similarly to our findings, due mostly to EOT (Ahrnberg et al. 2020). As better parental reflective functioning is related to better parental EA (Luyten et al. 2017), the link with EOT may indicate a role of reflective functioning. Furthermore, maternal EF has been linked to parental reflective functioning (Rutherford et al. 2018; Yatziv et al. 2020), and EF has been described to create space for parental mentalizing in caregiving situations (Rutherford et al. 2015). Future research should consider the role of parental mentalizing/reflective functioning specifically.

### Strengths and limitations

The main strength of this study is the integration of cognitive and personality research to further the understanding of parental caregiving. Another strength is the combination of multiple EF tasks into a composite, minimizing method variance. The EF tasks captured working memory and set-shifting, including visuospatial and learning elements. The findings might have differed if other EF tasks had been employed. Also, the tasks differ from caregiving situations, raising potential questions regarding ecological validity. However, considering how time-intensive EF data collection is, this study is a valuable addition to the research field, laying the foundation for future studies employing more ecologically valid EF measures.

The use of a self-report alexithymia measure entails the possibility for reporting biases. Furthermore, there have been indications that the TAS-20 subscale EOT has partly weak psychometric properties (Kooiman et al. 2002), and EOT has been found to have questionable internal consistency within the FinnBrain Birth Cohort (Kajanoja et al. 2017). On the other hand, EOT has been reported to show good construct validity, showing negative correlations with empathy, mind mindedness, and emotional intelligence (Bagby et al. 1994a; Grynberg et al. 2010; Parker et al. 2001). Although these limitations are noteworthy, the TAS-20 is to our knowledge the most suitable assessment method of alexithymia in contexts like the current study. The utilization of the EAS is a key study strength. Besides having sound psychometric properties (Biringen et al. 2014), this observation framework is utilized in clinical practice, extending the relevance of this study from research to a clinical context.

The main limitation of this study relates to sample characteristics. As only a general population sample was included, results should not be generalized to high-risk samples. The effect sizes were modest, and these weak associations are potentially related to sample characteristics. The participants reported a low prevalence of alexithymia, had normative EF levels, and exhibited mostly emotionally available caregiving behavior. This narrow variable variation might be reflected in the statistical associations. Exemplifying this dynamic, EF has been more strongly associated with emotional availability among mothers with substance use disorder (Porreca et al. 2018) than among general population mothers (Harris et al. 2021; Nordenswan et al. 2021). Compared to mothers with few risk factors, mothers who are at risk experience more negative parenting determinants that are more likely to strain their regulation capacity during caregiving. Further studies in varied populations are called for to clarify these dynamics. Furthermore, future research should include fathers/non-birthing parents, and parents with children of different ages.

## Conclusion

Our findings indicate an association between higher levels of maternal externally oriented thinking and lower emotional availability during early parenthood. If replicated, this implicates cognitive aspects of alexithymic traits as one of the individual parental determinants that shape caregiving behavior, which should be recognized within parenting interventions. This could be especially important among mothers with lower EF, as our results suggested that higher EF might buffer against such potential effects of alexithymic traits. Further studies with heterogeneous samples and at different developmental phases are needed to support and extend our results, and research on the role of parental mentalizing/reflective functioning in this context is especially needed.

## Electronic supplementary material

Below is the link to the electronic supplementary material.

Supplementary Material 1

## Data Availability

So far, the research group and collaborators have privileged access to the data over the rest of the research community, as the data set is exceptionally large, the onset of the cohort is relatively recent, and there are dozens of researchers involved ensuring active use of the data. Moreover, our informed consent does not include the permission to move the data to a shared database. However, the protocol for subsequent sharing of the data will be investigated and related permissions will be negotiated later on to enable as effective use of the unique cohort data as possible in the future.
