# Supplementary material for: Maternal alexithymia and caregiving behavior: the role of executive functioning - A FinnBrain Birth Cohort study
Source: Arch Womens Ment Health. 2024 Nov 5;28(1):67–75. doi: 10.1007/s00737-024-01523-4 (PMC11761824; doi:10.1007/s00737-024-01523-4)
Supplement: Supplementary file 1 — Supplementary Material 1 [file 737_2024_1523_MOESM1_ESM.docx]

**Online Resource 1**

**Materials and Methods**

**Participants**

The participants were drawn from the FinnBrain Birth Cohort Study (N = 3808 families; Karlsson et al. 2018). This prospective pregnancy cohort studies the combined influence of genetic and environmental factors on child development and later health outcomes (Karlsson et al. 2018). Expecting women eventually referred to give birth at Turku University Hospital in the Southwest Finland Hospital District and the Åland Islands in Finland were recruited along with their partners to the main FinnBrain cohort from 12/2011 to 4/2015. Recruitment took place through personal contact with a research nurse at gestational week 12, during free-of-charge ultrasound visits at maternal welfare clinics. Inclusion criteria were sufficient knowledge of Finnish or Swedish and a normal screening result. Of the 8895 newly pregnant women visiting the recruitment sites during the specified time period, 5790 were informed about the study. Of the women informed about the study, 66% decided to participate. The FinnBrain cohort population resembles the source population, with the exception that there is a lower prevalence of younger, smoking and multiparous women in the cohort compared to all deliveries at the Turku University Hospital from 2012 to 2014 (Karlsson et al. 2018).

The main data for this study was collected within a FinnBrain sub-study, The Child Development and Parental Functioning Lab. The current study’s mothers (N = 119) attended two separate visits for the assessment of maternal neurocognitive functioning and mother-child interaction, which were both conducted when the children were 2.5 years old. Recruitment to these visits was primarily based on participation in prior visits at the sub-study’s lab. From 2012 to 2013, mothers from the main FinnBrain cohort were randomly selected for recruitment to a study visit conducted during pregnancy, during which maternal cognitive functioning was assessed. Exclusion criteria were insufficient Finnish language skills and self-reported neurologic or psychiatric illness. Participants in this first maternal study visit during pregnancy (N = 247) were invited to follow-up assessments at 1 year and 2.5 years after delivery. During recruitment to the 2.5-year study visit, the recruitment list was expanded with mothers whose children had participated in a separate study visit assessing child self-regulation. Of the 198 mothers who completed the study visit assessing maternal cognitive functioning at 2.5 years after delivery, 137 had also attended a separate study visit together with their 2.5-year-old child, during which maternal caregiving behavior was assessed. Of these 137 mothers, 119 had additionally filled out an alexithymia questionnaire that was sent out to the whole cohort at six months postpartum. As data on maternal alexithymic traits, caregiving behavior, and cognitive functioning was required for inclusion in the present study, these 119 mothers formed the sample of the present study.

At 2.5 years after delivery, the 119 mothers participating in this study had a mean age of 34.17 years (*SD* = 4.71 years, range = 21.81 – 44.92 years). At this time, 41.2% of the mothers were primiparous, while 42.9% had two children, 12.6% had three children, 2.5% had four children, and 0.8% had five children. At two years after delivery, 79% of the participants filled out a couple relationship questionnaire, indicating that they were in a couple relationship at the time. This offers a rough estimation of the proportion of participants who were in a couple relationship at 2.5 years after delivery. Information about educational level, occupation and income level was gathered when the pregnant women were recruited to the main cohort, i.e. approx. three years prior to the collection of this study’s main variables of interest. At this time, almost half of the participants (45.4%) had a university level education, 29.4 % had a polytechnics education, and 25.2% had a high school/vocational education (<12 years). At the beginning of pregnancy, a majority of the mothers were employed (78.1%), while a few were unemployed (3.4%), students (5.9%), stay-at-home mothers (7.6%), or occupied otherwise (5.0%). After taxes, 28.6% of the participants had a monthly income of 1500€ or less, 59.3% had an income between 1501€ and 2500€, 9.3% had an income between 2501€ and 3500€, while 2.5% had an income over 3500€. At delivery, the 119 mothers in the current study had a slightly higher mean age (31.10 years) than the remaining 3689 mothers in the cohort (30.21 years), and this difference was significant (*t*[3806] = 2.04, *p* = .04). At this time, the participants in this study had also attained a significantly higher level of education than the remaining mothers in the whole sample (*X*^2^[2, *N* = 3078] =10.79, *p* = .01).

**Measures**

*Executive Functioning*

Cogstate task are computerized adaptions of standard neuropsychological tasks (Pietrzak et al. 2009). Of the 12 Cogstate tasks completed during the study visit, five tasks thought to tap onto EF were selected: Two-Back (TWOB), Set-Shifting (SETS), Groton Maze Learning (GML), Continuous Paired Associate Learning (CPAL), and International Shopping List (ISL). As any EF task engages both general EF as well as task-specific processes (Friedman and Miyake, 2017) the five EF tasks were combined into an EF composite to optimize reliability. In line with a previous factor analytic study (Nordenswan et al. 2020) the task outcome variables thought to best capture EF-related variance were utilized. More specifically, for the tasks with multiple test rounds, the result from the first test round was chosen instead of the summative score from all test rounds. This is because the novelty of a task, and thereby its EF load, is highest in the beginning. Cogstate completion and integrity pass rates were calculated, and classified nearly all scores as reliable measurements. The integrity pass rates and completion pass rates were 100% for GML, ISL and CPAL. Seven of the TWOB measurements were excluded from due to an insufficient result integrity, while one SETS score was excluded due to an insufficient task completion rate. The outcome variables were reversed for the Continuous Paired Associate Learning test and the Groton Maze Learning Test. Thus, a higher value equaled a better result for all EF tasks. The task scores were standardized, combined into an EF mean score, which was re-standardized. For the few participants with excluded TWOB or SETS scores, the EF composite was based on the mean value of the remaining task scores. Brief descriptions of the five Cogstate EF tasks are provided below. See Nordenswan et al. (2020) for more details about the tasks and their combination into an EF composite.

The Two Back Test (TWOB). This task is based on the n-back paradigm, and assesses working memory. In the TWOB, a playing card is shown at the center of the screen, and the participant is to decide whether it is the same card as the one shown two cards ago. The task terminates after 32 correct responses. The arcsine transformation of the square root of the proportion of correct responses was used as the outcome variable.

The Set-Shifting Test (SETS). This task assesses set-shifting ability, and is similar to the Wisconsin Card Sorting Test. In this task, a playing card is shown at the center of the screen, and the participant is to guess whether the card contains a target stimulus (i.e., a color or number). A sound indicates whether the response was correct, and the next stimulus is only displayed after the correct response has been made. In this way, the participant is taught the correct card dimension. After a while, the card dimension changes, and the new rule must be learnt to proceed. The task terminates after 120 correct responses. The arcsine transformation of the square root of the proportion of correct responses was used as the outcome variable.

The Groton Maze Learning Test (GML). This task is based on earlier hidden maze tasks. This task encompasses a notable visuospatial component. It taps on multiple and more complex aspects of EF, like working memory updating, planning and problem solving. The participant is first taught the task rules in a small practice grid. In the main task, the participant guesses a hidden, 28-step pathway from the top left corner to the bottom right corner of a 10 x 10 grid of tiles on the screen. Feedback is provided concerning the correctness of each guess. After an incorrect move, the participant must click on the last correct tile and then make a different choice. The task is repeated 5 times, with the same pathway. The number of errors during the first test round (i.e., the round after the first learning trial) was employed as the outcome variable.

The Continuous Paired Associate Learning Test (CPAL). This task is based on the visual paired associate learning paradigm. The CPAL measures the ability memorize sets of associations between spatial locations and simple patterns, so that later exposure to one aspect of that same information stimulates recall of the other. The participant is first taught the task rules with two practice figures. In the actual task, the participant is taught where eight differently shaped and colored figures are located on the screen, which are covered by neutrally colored circles. Two additional, empty circles are also present on the screen. As the figures are one at a time shown at the center of the screen, the participant is to click on the circle under which the figure is hidden. Incorrect responses result in an error sound, and the correct response is required to proceed. During the six test rounds, the figures are presented in varied orders. The number of errors during the first test round (i.e., the round after the first learning trial) was employed as the outcome variable.

The International Shopping List Test (ISL). This task is a verbal list learning task, i.e., a neuropsychological measure frequently employed to assess verbal learning and memory. In the ISL, the participant listens to a shopping list of 12 items that is read aloud, and then repeats the recalled items. The same process, with the same shopping list, is repeated three times. The number of correct responses from the first test round was utilized as the outcome variable.

*Alexithymic Traits*

The Toronto Alexithymia Scale (TAS-20) is self-report questionnaire. It is one of the most commonly used measures of alexithymic traits, and is viewed as a valid and reliable measure of alexithymia (Bagby et al. 2020). A validated Finnish-language version of the questionnaire is available (Joukamaa et al. 2001). TAS-20 items are rated on a 5-point Likert scale, the total score ranges from 20 to 100, and higher values reflect more alexithymic traits. TAS-20 total scores exceeding 60 points are taken to indicate a high level of alexithymia, while total scores from 52 to 59 are considered to reflect a moderate level of alexithymia (Taylor et al. 1997). Besides the alexithymia sum score, the TAS-20 encompasses three subscales: Difficulty Identifying Feelings (DIF), Difficulty Describing Feelings (DDF), and Externally Oriented Thinking (EOT). As the TAS-20 subscales have previously been found to associate differently with caregiving behavior (Ahrnberg et al. 2021), we included both the TAS-20 sum score and the subscales in the analyses. Prior to the inclusion in the EOTxEF interaction term, the EOT subscale was standardized.

*Caregiving Behavior*

The Emotional Availability Scales (EAS; Biringen 2008), operationalizes a parent-child dyad’s capacity to share an emotionally healthy relationship (Biringen et al. 2014). The EAS have been extensively studied, and are considered a sensitive and valid measure of dyadic relational affective quality (Biringen et al. 2014). The EAS include four dimensions of parental caregiving behavior. *Sensitivity* implicates the ability to be authentically and positively emotionally present during caregiving situations, as well as appropriately interpreting and flexibly reacting to the child’s emotional cues. *Structuring* refers to the capacity to mentor the child’s pursuits, while also strengthening the child’s sense of autonomy. *Non-Intrusiveness* describes the ability to be available to the child without being intrusive, i.e., overstimulating, overprotecting, overdirecting, or interfering. *Non-Hostility* implicates the absence of threatening, hostile, or frightening behavior, and the ability to create a non-hostile emotional climate for the child. These dimensions are scored from 1 to 7 on a 14-point Likert scale, with higher scores equaling better EA. Scores from 1 to 2 are considered highly problematic, scores from 2.5 to 3.5 indicate detachment in the relationship, scores from 4 to 5 are viewed as somewhat problematic and indicate complicated EA, while scores from 5.5 to 7 are indicative of healthy EA in the relationship (Biringen and Easterbrooks 2012) The coding was conducted by three coders, who had received training and a certificate of reliability from the developer of the EAS. Interrater-reliability was checked for 1/10th of all the free-play episodes conducted when the children were 2.5 years old (N = 474). Divergent coding was negotiated between the coders. The intraclass correlation coefficient for sensitivity ranged from 0.83 to 0.91, for structuring from 0.84 to 0.91, for non-intrusiveness from 0.84 to 0.90, and for non-hostility from 0.70 to 0.85. In the present study, the four parental EAS dimensions were combined into an EA mean score. Higher values on this EA composite describes better EA, i.e., more sensitivity, more structuring, less intrusiveness, and/or less hostility.

**References**

Ahrnberg, H., Korja, R., Scheinin, N. M., Nolvi, S., Kataja, E.-L., Kajanoja, J., … Karukivi, M. (2021). Maternal Alexithymic Traits Are Related to Lower Maternal Sensitivity and Higher Hostility in Maternal Caregiving Behavior-The FinnBrain Birth Cohort Study. *Frontiers in Psychology, 12*, 704036–704036. <https://doi.org/10.3389/fpsyg.2021.704036>

Bagby, R. M., Parker, J. D. A., & Taylor, G. J. (2020). Twenty-five years with the 20-item Toronto Alexithymia Scale. *Journal of Psychosomatic Research*, *131*, 109940–109940. <https://doi.org/10.1016/j.jpsychores.2020.109940>

Biringen, Z. (2008). *The Emotional Availability (EA) Scales Manual,* 4^th^ Edn. Boulder, CO: International Center for Excellence in Emotional Availability.

Biringen, Z., Derscheid, D., Vliegen, N., Closson, L., & Easterbrooks, M. A. (2014). Emotional availability (EA): Theoretical background, empirical research using the EA Scales, and clinical applications. *Developmental Review*, *34*(2), 114–167. <https://doi.org/10.1016/j.dr.2014.01.002>

Biringen, Z., & Easterbrooks, M. A. (2012). Emotional availability: Concept, research, and window on developmental psychopathology. *Development and Psychopathology*, *24*(1), 1–8. <https://doi.org/10.1017/S0954579411000617>

Friedman, N. P., & Miyake, A. (2017). Unity and diversity of executive functions: Individual differences as a window on cognitive structure. *Cortex, 86*, 186–204. <https://doi.org/10.1016/j.cortex.2016.04.023>

Joukamaa, M., Miettunen, J., Kokkonen, P., Koskinen, M., Julkunen, J., Kauhanen, J., Jokelainen, J., Veijola, J., Läksy, K., & Järvelin, M. R. (2001). Psychometric properties of the Finnish 20-item Toronto Alexithymia Scale. *Nordic Journal of Psychiatry*, *55*(2), 123–127. https://doi.org/10.1080/08039480151108561

Nordenswan, E., Kataja, E.-L., Deater-Deckard, K., Korja, R., Karrasch, M., Laine, M., … Karlsson, H. (2020). Latent Structure of Executive Functioning/Learning Tasks in the CogState Computerized Battery. *SAGE Open*, *10*(3), 215824402094884-. <https://doi.org/10.1177/2158244020948846>

Pietrzak, R. H., Maruff, P., & Snyder, P. J. (2009). Convergent validity and effect of instruction modification on the groton maze learning test: a new measure of spatial working memory and error monitoring. *The International Journal of Neuroscience*, *119*(8), 1137–1149. <https://doi.org/10.1080/00207450902841269>

Taylor, G. J., Bagby, R. M., & Parker, J. D. A. (1997). *Disorders of affect regulation: alexithymia in medical and psychiatric illness*. Cambridge: Cambridge University Press.
